# Lead-Wire-Resistance Compensation Technique Using a Single Zener Diode for Two-Wire Resistance Temperature Detectors (RTDs)

**DOI:** 10.3390/s20092742

**Published:** 2020-05-11

**Authors:** Wei Li, Shusheng Xiong, Xiaojun Zhou

**Affiliations:** 1 School of Mechanical Engineering, Zhejiang University, Hangzhou 310027, China; 11725081@zju.edu.cn (W.L.); me_zhouxj@zju.edu.cn (X.Z.); 2 College of Energy Engineering, Zhejiang University, Hangzhou 310027, China

**Keywords:** RTD, zener diode, temperature measurement, lead wire

## Abstract

In remote measurement systems, the lead wire resistance of the resistance sensor will produce a large measurement error. In order to ensure the accuracy of remote measurement, a novel lead-wire-resistance compensation technique is proposed, which is suitable for a two-wire resistance temperature detector. By connecting a zener diode in parallel with the resistance temperature detector (RTD) and an interface circuit specially designed for it, the lead-wire-resistance value can be accurately measured by virtue of the constant voltage characteristic of the zener diode when reverse breakdown occurs, and compensation can thereby be made when calculating the resistance of RTD. Through simulation verification and practical circuit testing, when the sensor resistance is in 848–2120 Ω scope and the lead wire resistance is less than 50 Ω, the proposed technology can ensure the measuring error of the sensor resistance within ±1 Ω and the temperature measurement error within ±0.3 °C for RTDs performing 1000 Ω at 0 °C. Therefore, this method is able to accurately compensate the measurement error caused by the lead wire resistance in two-wire RTDsand is suitable for most applications.

## 1. Introduction

High-precision temperature measurement provides basic data for product development and industrial automation applications to improve product quality and ensure production safety. Due to its excellent linearity, measurement repeatability and stability [1], a resistance temperature detector (RTD) is widely used therein. However, for remote measurements, the electrical resistances of long connecting lead wires between the RTD and the control room instrument produce an appreciable error in measurement. This unwanted error varies not only with the length of the lead wires but also with ambient temperature variations where the lead wires layout [2]. Therefore, methods to reduce or eliminate measurement errors caused by lead wire resistance have been studied in many literatures.

Currently, the aforementioned problem is addressed by adding lead wires. For example, in Reference [3,4] three-wire and four-wire techniques were used separately. However, the cost of one additional lead wire in a three-wire RTD and of two additional lead wires in a four-wire RTD will be extremely high and with extra difficulty of wiring, especially in industries where a large number of process points are to be monitored from a control room located at a remote place [5], such as chemical, thermal power, electric plant and other industries. In the bridge-based measuring system, lead wire resistance also exerts an adverse effect on measurements. Authors in Reference [6,7] presented a method to eliminate lead wire resistance for quarter- and half-bridge interface circuit respectively, which employed several operational amplifiers (OPAMPs) and made use of their high input impedance. This method was able to eliminate the unfavorable effect on the measurement caused by lead wires, but it cannot obtain the specific lead-wire-resistance values, in addition, its interface circuits and power circuits were complicated due to the OPAMPs. Similar methods using OPAMPs were also published in Reference [8,9,10]. All of these bridge-based interface circuits used a three- or four-wire method, except for Reference [6].

Therefore, researches have been carried out on the compensation method of two-wire sensors. In Reference [11], a compensation resistor is selected having the same value and of the same material of the lead resistances combined with three operational amplifiers and a constant current source to compensate the lead wires error. Although the circuit of this method is simple, the compensation resistance must be adjusted manually if the lead wire length is changed due to reconstruction. In addition, the compensation effect due to temperature drift of the lead wire resistance remains to be validated. The authors of [5] proposed a novel lead-wire-resistance compensation technique using a two-wire RTD. This technique employed two diodes, one of which was in series with the RTD and the other was in parallel with the series circuit composed of the RTD and the first diode. A current source, four analogy switches and four sample-holding circuit were used in its interface circuit, and an operational amplifier is used to output the voltage with respect to the RTD resistance. In principle, this technique completely eliminated the measurement error caused by the lead wire resistance and its temperature drift, but its measurement accuracy depended on the consistency of the forward voltage drop of the two diodes. Therefore, in order to achieve a high precision measurement, it is indispensable to measure and pair the diodes in advance. Besides, this technique cannot obtain the true value of the lead wire resistance. This method was also seen in other references, and some improvements had been made in the interface circuit. In the Reference [12], three single-pole double-throw (SPDT) analog switches are used to realize the lead-wire-resistance compensation, which was independent of the voltage reference. The authors of [13,14] used a voltage-to-current converter to provide the current and reduced the number of SPDT analog switches to two. A Chinese patent invented a method that employed the transient characteristics of resistor–capacitor (RC) circuit to measure the lead wire resistance and compensate measurement error of the thermal resistance. In this method, the measurement precision is not affected by the characteristics of the additional capacitor, and the lead-wire-resistance value could be obtained in real time, which avoided the negative influence of the temperature drift of the lead wire resistance. According to the patent, the update rate is less than 5 Hz, and it is not suitable for the applications where temperature changes need to be tested dynamically. In References [15,16], the interface circuits for four-wire resistive sensor were designed by the similar method of multiple charge and discharge to a capacitor, and the update rate reported was 25 Hz. The authors in References [17,18] combined the method for two-wire sensors reported in Reference [5] and also proposed the method using a capacitor, and the their minimum complete measurement cycle required 5.3 ms. These methods have the same problem of limited update rate.

The aim of this paper is to solve the problem of lead-wire-resistance compensation for two-wire RTDs based on as simple interface circuit as possible, on the premises that the measurement accuracy and the update rate are acceptable. So that, a new technique for compensating the lead-wire-resistance measurement error of two-wire RTD is proposed, which can also resist the negative influence of the temperature drift of the lead wire resistance on the compensation circuit. This technique only adds one zener diode on the sensor side, and the lead wire resistance and RTD resistance can be accurately measured through time sharing based on the stability of the reverse breakdown voltage and the minuteness of reverse leakage current. A simple implementation and its validation of the interface circuit will be described later in this paper, which only requires one SPDT analog switch and a pair of reference power sources, and OPAMPs are not required. Compared to the current methods, the proposed technique not only uses fewer components, only one zener diode, for two-wire RTDs, but also can detect and diagnose the failures of the lead wires by measuring the lead wire resistance in real time.

## 2. Materials and Methods

### 2.1. Principle

**Figure 1:**
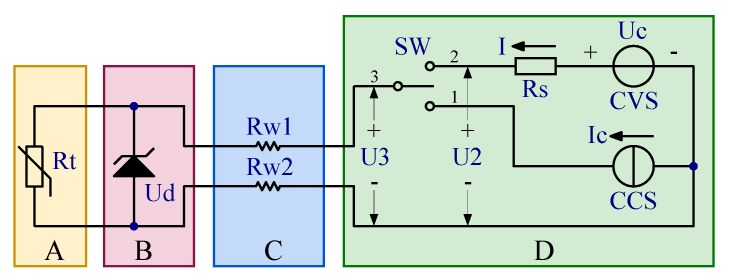
Circuit schematic for the lead-wire-resistance compensation technique.

Combined with the engineering application scenarios of remote measurement, the circuit schematic of the technique proposed in this paper is shown in Figure 1 which is composed of four regions. R_t_ represents an RTD, and Region A is the region of the object to be measured. The area B where the zener diode is located should be as close to the RTD as possible, and the ambient temperature in this area should not change significantly due to the change of the temperature in Region A. The RTD in Region A and zener diode in Region B are connected by two wires. As their length is very limited, the resistance of these wires is negligible. Region C represents the layout path of the lead wires. In the remote measurement system, the path is quite long. Its specific length depends on the practical engineering conditions and is often changed due to engineering changes and other reasons. The resistances R_w1_ and R_w2_ represent the lead wire resistance, and it is generally believed that R_w1_ = R_w2_ = R_w_. Region D represents the remote-control room or equipment room, where the interface circuit is located. The other elements in Figure 1 will be described combined with the measurement procedures in the next paragraph.

In the proposed technique, a complete measurement procedure of RTD resistance consists of three steps:

1. Measurement of lead wire resistance.

Switch SW in Region D of Figure 1 is switched to Position 1. Constant current source (CCS) provides current I_c_ through lead wire resistors R_w1_ and R_w2_, giving rise to the reverse breakdown of the zener diode and establishing a stable voltage U_d_ across it. Since the resistance of zener diode after reverse breakdown is very small, most of the current goes through the diode. Although the current flowing through R_t_ is relatively small, the negative influence of RTD’s self-heating effect on the temperature measurement must be considered. The voltage at Position 3 marked as U_3_ is measured. Then the lead wire resistance R_w_ can be calculated by substituting U_3_ into Equation (1).

(1) $$U_{3}=2I_{c}\cdot R_{w}+U_{d}$$

Record the values for U_3_ and R_w_, which will be used in the following steps.

2. Measurement of working current.

SW is switched to Position 2. RTD is supplied by constant voltage source (CVS) with a voltage of U_c_, which is not more than U_d_. At this time, the zener diode works in the reverse cut-off mode, and the equivalent resistance is very large. The current passing through it marked as I_d_ is in microamperes range [19], whose specific value should be obtained by measurement in advance. In this step, the voltage at Position 3 is marked as U_3_^’^, which is measured and satisfies Equation (2).

(2) $${U_{3}}^{’}=2I\cdot R_{w}+\left(I-I_{d}\right)R_{t}$$

The I in Equation (2) represents the output current of the CVS. At the same time, the voltage measured at Position 2 is represented by U2. Thus, I can be obtained from Equation (3). Save the values of U_2_ and U_3_^’^ for Step 3:

(3) $$I\;=\;\left(U_{c\;}-{\;U}_{2}\right)/R_{s}$$

where R_s_ represents a calibration resistor.

3. Calculation of RTD resistance.

By substituting Equations (1) and (3) into Equation (2), the resistance of RTD can be obtained, as shown in Equation (4).

(4) $$R_{t}=\frac{I_{c}\cdot R_{s}\cdot {U_{3}}^{’}+\left(U_{C}-U_{2}\right)\left(U_{d}-U_{3}\right)}{I_{c}\left(U_{c}-U_{2}-I_{d}\cdot R_{s}\right)}$$

Equation (4) shows that it is a simple algebraic equation, which can be solved in a microcontroller to obtain RTD resistance. Then the RTD resistance can be converted to the temperature simply by look-up table method or directly computation of the polynomial.

From Equation (4), it can be seen that the factors affecting the measurement accuracy of RTD resistance are as follows:

- Stability of U_d_ and I_d_
- Stability of U_c_ and I_c_
- Measurement accuracy of U_2_, U_3_ and U_3_^’^
- Accuracy and Stability of R_s_

The first one is the key and most difficult factor to implementing the proposed technique. Other factors can be met by conventional technical means, such as using high-precision analog to digital sampling, low temperature drift power source and sampling resistance.

Compared with the I-V curve of normal diode, the I-V curve of zener diode has a narrower breakdown voltage range and a larger curve slope. Therefore, the change of the reverse breakdown voltage of U_d_ with its operating current is very small. At present, for high-precision zener diodes, the nominal error of the reverse breakdown voltage can reach 0.05%, and the device consistency is very high. Besides, the reverse leakage current of the zener diode can be kept relatively stable in the reverse cutoff region. These characteristics of zener diode make it possible to calibrate the system. In order to obtain higher measurement accuracy, it is necessary to calibrate the values of reverse breakdown voltage and reverse leakage current of zener diode in each measurement circuit and store them in the nonvolatile memory.

Based on this principle, the update rate depends on the response time of zener diode under the step voltage input (200 μs), the single switching time of the analog switch (18 ns), the two analog to digital sampling times and the calculation time of the micro controller. The values in brackets above are from the device datasheet used in the circuit prototype in Section 2.3. The conversion time of conventional 16-bit analog to digital chips is 2 μs, and the calculation time of Equation (4) for 32-bit microcontroller with 72 MHz should not exceed 30 μs. Therefore, the total time for one measurement should be within 250 μs. In other words, the update rate in this paper can reach 4 KHz.

### 2.2. Simulation

The circuit model as shown in Figure 1 was established in the circuit simulation software Multisim 14. The model simulates the resistance changes of RTD and lead wires through adjustable resistor. In this paper, RTD with the nominal value of 1000 Ω at 0 °C was focused on, and the range form −38.67 °C to +299.86 °C was selected as the research range. The range of its resistance is 848 Ω–2120 Ω accordingly [20]. Providing a maximum of 50 Ω of single lead wire, the distance is about 553 m when a shielded tin-plated copper conductor with nominal cross section of 0.2 mm^2^ was selected, which can cover most practical engineering application. The parameters of the simulation model are shown in Table 1.

The selection of CCS current takes two factors into consideration: On one hand, in order to reduce the self-heating effect of RTD, the working current should be as small as possible. On the other, because the greater the CCS current is, the higher the stability of the voltage across the zener diode is according to the simulation results, in order to ensure the measurement accuracy, the voltage should be as stable as possible and the CCS current should be as large as possible. 

According to multiple simulation results, when the CCS current was selected as 10 mA, the change of voltage across the zener diode and the current through RTD are both appropriately small as shown in the following paragraphs. It not only guarantees the sufficient measurement accuracy, but also avoids the serious self-heating effect.

If different types of zener diodes are used, the stability of the voltage shall be re-measured and converted it into measurement accuracy. Select the CCS current as small as possible within the allowable measurement accuracy.

The specific procedures of simulation are shown in the following paragraphs.

At first, Step 1 in Section 2.1 is simulated to study the stability of U_d_ when the resistance of RTD and lead wire is changing under CCS supply. The simulation results are shown in Table 2. It shows that the voltage variation across zener diode in the research range is 0.006 V at 10mA current, which is 0.25% of its nominal value of breakdown voltage. 

**Figure 2:**
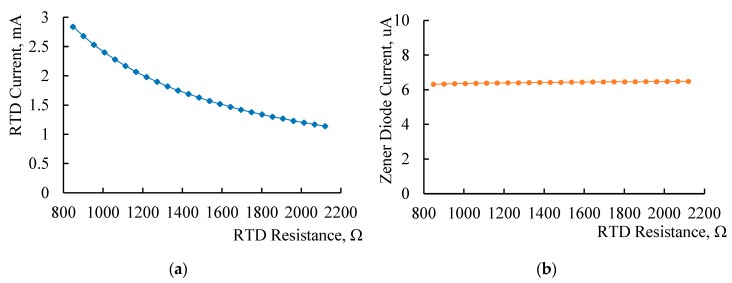
(**a**) Current through RTD changes over its resistance under CCS supply; (**b**) current through zener diode changes over the resistance of RTD under CCS supply.

Then, simulation results show that the current through RTD changes with its resistance value, as shown in Figure 2a. In the research range, the current gradually decreased from 2.88 mA to 1.15 mA. Although the RTD resistance is not measured in Step 1, too much current will produce self-heating effect and affect the accuracy of temperature measurement, which must be considered. For instance, the technique of measurement under pulse excitation can be adopted to avoid the self-heating effect [21].

After that, the switch SW is switched to Position 3 to simulate Step 2 in Section 2.1. The reverse leakage current of zener diode should be paid more attention to evaluate its effect on the measurement accuracy. Figure 2b shows the change of the reverse leakage current of zener diode in RTD research range when lead wire resistance is fixed to 15 Ω. It is basically stable between 6 and 7 μA. Simulation results show that further when the lead wire resistance changes between 0–50 Ω, the change of reverse leakage current of zener diode is less than 0.1 μA. The stability of this value guarantees the feasibility of obtaining RTD resistance from Equation (4) in aforementioned Step 3.

In the end, RTD resistance is calculated according to Equation (4) combined with the parameters in Table 1, where U_d_ is 2.413 V and I_d_ is 6.40 μA. The resistance is calculated as temperature according to the polynomial in Reference [20], and the result is shown in Table 3.

Table 3 shows the RTD resistance figured out according to Equation (4) at thirteen set points by arithmetic sequence and five different lead wire resistances in the research range. The comparison between the set points and the calculation values indicates that the largest error of RTD resistance is +2.04 Ω and −0.68 Ω, the maximal error of the temperature is +0.52 °C and −0.19 °C.

The simulation results show that the technique can effectively compensate the adverse effect of the lead wire resistance within 50 Ω on the temperature measurement. Meanwhile, the accuracy of temperature measurement reaches ±0.52 °C or 0.3% of research range.

### 2.3. Experiment

**Figure 3:**
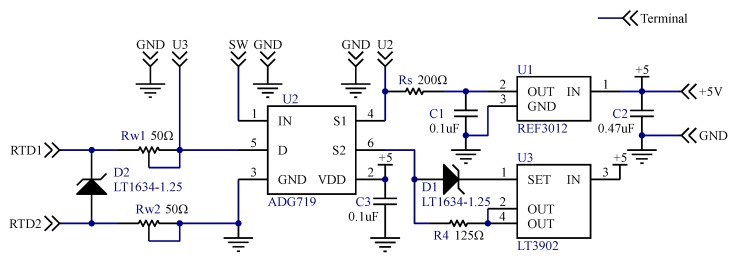
Schematic of RTD interface circuit.

**Figure 4:**
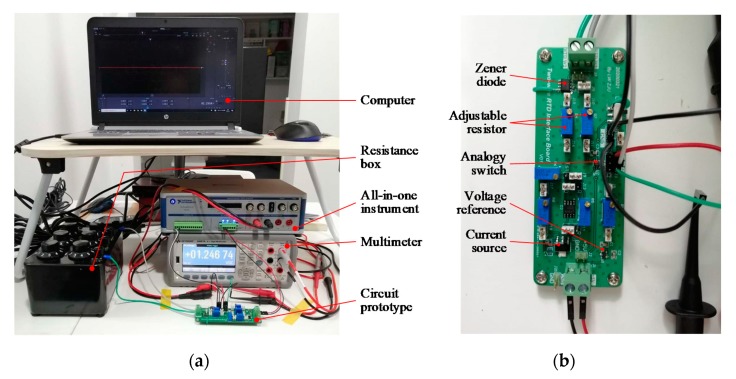
(**a**) General view of the experimental devices; (**b**) close-up shot of the circuit prototype.

Based on the feasibility of the above simulation, to implement validation experiment, the schematic of the RTD interface circuit was designed as shown in Figure 3 and its practical circuit prototype was fabricated as shown in Figure 4b.

The lead wire resistance was simulated by precise adjustable resistors R_w1_ and R_w2_. RTD was simulated by a resistance box, the accuracy of that is 0.1%. The circuit employed a high-precision zener diode (LT1634; Linear Technology Corp., Milpitas, CA, USA), and was excited by +5 V single power source. A current source chip (LT3902; Linear Technology Corp., Milpitas, CA, USA) and voltage reference chip (REF3012, Texas Instruments Inc., Dallas, TX, USA) integrated into the circuit to build the power supply circuit. Circuit switching realized by an analog switch (ADG719; Analog Devices Inc., Norwood, MA, USA). The photo of the circuit prototype was shown in Figure 4b.

The devices used in the experiment were shown in Figure 4a, which included: (1) a 6.5 bit digital multimeter (34461A; Keysight Technologies, Inc., Loveland, CO, USA) for voltage and current measurement; (2) an all-in-one instrument (VB8012; National Instruments Corp., Austin, TX, USA) with a laptop working as digital output interface, adjustable voltage source and 5.5 bit digital multimeter; (3) A rotary resistance box and (4) the interface circuit prototype fabricated according to Figure 3.

The electrical connection between the experimental devices is as follows: terminals of U_2_ and GND nearby in Figure 3 were connected to the voltage measurement channels of multimeter 34461A; terminals of U_3_ and GND nearby were connected to the voltage measurement channel of the all-in-one instrument VB8012; terminal SW is connected to the digital output port of VB8012; terminals of +5 V and GND nearby were connected to the power output port of VB8012; terminals of RTD1 and RTD2 were connected to the resistance box.

At the beginning of the experiment, the values of R_s_, U_d_, U_c_ and I_c_ were measured one by one through the multimeter 34461A. They were recorded and would be used in the subsequent calculation.

The experiment followed the following steps:

1. Set the values of the resistance box for RTD and adjustable resistors for the lead wires.
2. Enable the digital output port of VB8012 to be high to switch on the CCS and shut off the CVS.
3. Measure and record the voltage of U_3_ by VB8012.
4. Set the digital output port of VB8012 to be low to switch on the CVS and shut off the CCS.
5. Measure and record the voltage of U_3_^’^ by VB8012 and the voltage of U_2_ by 34461A.

After alternating the set points of the resistance box and the adjustable resistors, the above steps were repeated one time to obtain a second set of measurements. After many repetitions, more measurements were obtained. When the measurement was completed, all the data was input into a spreadsheet to calculate the measured value of the resistance box and the corresponding temperature.

## 3. Results and Discussion

All experiments were carried out at an ambient temperature of 17.5 °C. The measured values of other constants required for calculation are shown in Table 4.

**Figure 5:**
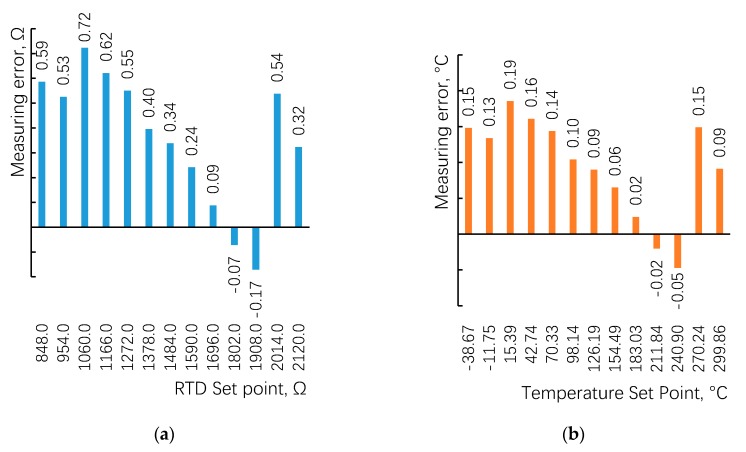
(**a**) Measurement errors of resistance when lead wire resistance is 1 Ω; (**b**) Measurement errors of corresponding temperature when lead wire resistance is 1 Ω.

When the lead wire resistance was set to 1 Ω, under the thirteen set points of RTD resistance from 848 Ω to 2120 Ω, the values of resistance box were measured by the proposed technique, and was translated into corresponding temperature by the polynomial in Reference [20]. The error statistics of resistance and temperature were obtained by comparing with the set points, as shown in Figure 5. 

**Figure 6:**
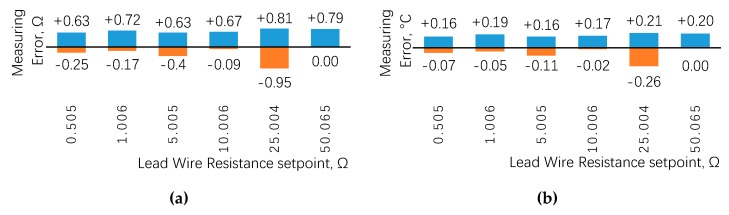
(**a**) Measurement errors of resistance under different lead wire resistance; (**b**) measurement errors of temperature under different lead wire resistance.

Figure 5a shows that the measurement error range of resistance is −0.17 Ω–+0.72 Ω, the deviation for corresponding temperature is −0.05 °C–+0.19 °C, as shown in Figure 5b. Figure 5 shows that the maximum error of the measurement appeared in the set point of 1060 Ω or 15.39 °C. Further inspection of the experiment data under other lead wire resistances given in Figure 6 reveals the same pattern.

By changing the adjustable resistance, the lead wire resistance was set to the values shown in Figure 6. Then, the thirteen set points of RTD resistance as shown in Figure 5a were measured one by one, and the maximum errors of resistance and temperature were taken and summarized in Figure 6a,b, in which the blue column represents the maximum positive error and the orange column represents the maximum negative error. Figure 6 indicates that the all errors do not exceed ±0.3 °C among the six groups of lead wire resistance, and the maximum values are +0.21 °C and −0.26 °C. The error distribution has no specific relationship with the lead wire resistance.

Experiment results shows that the lead-wire-resistance compensation technique proposed in this paper can accurately measure the RTD resistance when the lead wire resistance range is 0.5–50 Ω. In the measuring range of 848–2120 Ω or −38.67 °C–+299.86 °C, the measurement error of temperature is within ±0.3 °C according to the temperature characteristic polynomial of RTDs performing 1000 Ω at 0 °C. This indicates that the proposed technique can effectively compensate RTD lead wire resistance, and the compensation accuracy can meet the requirements of most remote temperature measurement using RTDs.

Besides being able to achieve lead-wire-resistance compensation for two-wire sensors, the benefits of this technique include: The component used is simple and small in size for only one zener diode is paralleled with the sensor; the time for measuring is short, because the working point of the zener diode can be established in a very short duration, the update rate of the sensor that is achievable is much higher compared to the scheme with the capacitor being charged and discharged; the consistency of the reverse breakdown voltage among the zener diodes is excellent, and its initial precision of 0.05% is available; the specific resistance of lead wires can be acquired in real time, which renders fault diagnosis of lead wire feasible.

The proposed technique can be popularized to other applications of remote measurement using resistive sensors with lead wires such as resistive displacement sensors, magnetic field sensors, piezoresistive sensors, etc. For example, the strain gauge described in Reference [22] and the flow sensor mentioned in Reference [23] both need to eliminate the errors caused by lead wire resistance through four-wire configurations. So that these sensors are able to turn into two-wire system from three- or four-wire system, saving significant cost of lead wire and time for construction.

## 4. Conclusions

A new lead-wire-resistance compensation technique for two-wire resistive temperature sensor was described in this paper. The principle is proved to be feasible by circuit simulation and that the feasibility is verified by experiment. Firstly, the lead-wire-resistance compensation equation was derived based on the circuit principle. Then the components selection and circuit simulation were implemented in Multisim 14 to evaluate the measurement error and verify the feasibility of the technique. Finally, a prototype of interface circuit for experiment was fabricated. The experiment system was setup using a standard resistance box to simulate the RTD and with the help of a digital multimeter and an all-in-one instrument. The measuring values of the resistance box were obtained, and the temperature errors were figured out. The results show that the proposed technique fulfills RTD resistance measurement within the research range in this paper, and the temperature measurement error is less than ±0.3 °C or the percent error is better than 0.14%.

## Figures and Tables

**Table 1:** Parameters used in the simulation model.

Parameters	Breakdown Voltage of Zener Diode	Output Current of CCS	Output Voltage of CVS	On-State Resistance of Analog Switch	**Temperature**
Value	2.4 V	10.00 mA	1.250 V	0.5 Ω	27 °C

**Table 2:** Voltage across zener diode by the resistance of the resistance temperature detector (RTD) and lead wire (in V).

**Set Points of RTD Resistance (** **Ω** **)**		848.0	1060.0	1272.0	1484.0	1696.0	1908.0	2120.0
**Set Points of Lead Wire Resistance (** **Ω** **)**	0.5	2.410	2.412	2.414	2.415	2.415	2.416	2.416
	5	2.410	2.412	2.414	2.415	2.415	2.416	2.416
	20	2.410	2.412	2.414	2.415	2.415	2.416	2.416
	50	2.410	2.412	2.414	2.415	2.415	2.416	2.416

**Table 3:** Comparison of set points and simulation values about RTD resistance and corresponding temperature.

Number			1	2	3	4	5	6	7	8	9	10	11	12	13
**Set Points**	**Resistance/Ω**		848.00	954.00	1060.00	1166.00	1272.00	1378.00	1484.00	1590.00	1696.00	1802.00	1908.00	2014.00	2120.00
	**Temperature/°C**		−38.67	−11.75	15.39	42.74	70.33	98.14	126.19	154.49	183.03	211.84	240.90	270.24	299.86
**Observed Values**	R_w_ =0.5 Ω	Res./Ω	849.00	955.03	1061.01	1166.99	1272.96	1378.94	1484.90	1590.87	1696.82	1802.78	1908.73	2014.69	2120.62
		Temp./°C	−38.42	−11.49	15.65	43.00	70.58	98.39	126.43	154.72	183.26	212.05	241.10	270.43	300.03
	R_w_ =1 Ω	Res./Ω	850.04	956.03	1062.02	1168.00	1273.97	1379.95	1485.91	1591.87	1697.83	1803.79	1909.75	2015.69	2121.64
		Temp./°C	−38.15	−11.23	15.91	43.26	70.84	98.65	126.70	154.99	183.53	212.32	241.38	270.71	300.32
	R_w_ =5 Ω	Res./Ω	848.36	954.22	1060.11	1166.01	1271.92	1377.84	1483.76	1589.68	1695.61	1801.53	1907.45	2013.39	2119.32
		Temp./°C	−38.58	−11.69	15.41	42.75	70.31	98.10	126.13	154.40	182.93	211.71	240.75	270.07	299.66
	R_w_ =20 Ω	Res./Ω	848.39	954.25	1060.14	1166.05	1271.96	1377.87	1483.78	1589.73	1695.66	1801.58	1907.52	2013.44	2119.37
		Temp./°C	−38.57	−11.68	15.42	42.76	70.32	98.11	126.13	154.41	182.94	211.72	240.77	270.08	299.68
	R_w_ =50 Ω	Res./Ω	848.44	954.31	1060.20	1166.11	1272.03	1377.95	1483.89	1589.82	1695.75	1801.68	1907.62	2013.56	2119.49
		Temp./°C	−38.56	−11.67	15.44	42.77	70.33	98.13	126.16	154.44	182.97	211.75	240.80	270.12	299.71

**Table 4:** Parameters used in the simulation model.

Parameters	Reference Voltage of CVS	Output Current of CCS	Voltage Drop of Zener Diode	Leakage Current of Zener Diode	Sampling Resistance
Value	1.2468 V	10.005 mA	1.2514 V	1.75 μA	200.03 Ω
